# Resolutions of the Convention of Medical Teachers

**Published:** 1867-05

**Authors:** 


### Editorial Department.

### Resolutions of the Convention of Medical Teachers.

The space occupied by report of the proceedings of the American Medical Association, and of the Convention of Teachers of Medical Colleges, prevents extended remark upon the action of either of these bodies, though every member of the profession will feel a deep interest in both.

The subjects presented for the consideration of the Convention of Teachers, have been for many years under consideration, but no definite and positive results have ever been attained. One of the first objects proposed in the organization of the American Medical Association was, "that a uniform and elevated standard of requirements for the degree of M. D., should be adopted by all the Medical Schools in the United States;" and it was resolved that young men, before being received as students of medicine, should have acquired a suitable preliminary education. This general question has been under consideration for a long period; but from inherent difficulties and differences, from local and other interests, no definite and harmonious action has resulted. It cannot be expected that so great changes can be proposed without differences of opinion, in many respects—while in some of the changes proposed, all will agree. That medical students should have acquired preliminary education before being matriculated in a medical college, should attend three terms of Lectures, and pursue the study of medicine four years, are changes which can hardly be opposed by those who have the good of the profession in view. That the terms of Medical Lectures have been too short, and that too much has been expected of students during these terms, is also quite obvious. Six and sometimes seven lectures daily, of an hour each, have proved more than most students could listen to with advantage; and there can be no doubt that four lectures thoroughly digested would be more instructive than six, which it would generally be too tiresome to attend. Extension of Lecture term, then, will be a kindness and an advantage to medical students. Upon the point of what shall be taught before graduation, and what left to be attained after, great differences may be expected to prevail. That department of medicine which receives the attention of a teacher, is apt to rise in his estimation, to the highest rank, and be the most important in character. and first to be obtained. That the field has grown wider, and much more is now to be taught than was formerly necessary or possible, cannot be doubted; and no one but will see a propriety and an advantage in suitable provision for full instruction in all the general divisions of medical knowledge. The propositions presented to the American Medical Association and adopted quite unanimously by the Convention of Medical Colleges, merit the approval of the profession; and it cannot be doubted that medical schools will conform in letter and spirit as fully and as early as possible. Public sentiment in medicine is as powerful as elsewhere, and will favor those who cheerfully accept its reasonable demands. It was proposed to introduce a Professorship of Medical Ethics; evident, in the view of the author, that physicians were deficient in the common civilities and courtesies of life. If the first proposition is adopted, and students are required to be preliminarily educated, the "medical ethics" will hardly require to be specially taught. I have scarcely known cultivated physicians misuse each other; it comes of ignorance, uncultivated and uneducated barbarity. Medical Ethics requires attention, but cannot be reduced to arbitrary rules and regulations. Regard for the feelings and interests of others in the same profession, is a natural element in the composition of an educated gentleman; and if a physician has any appreciable amount of the better qualities of humanity, his ethics will be unobjectionable.

Other propositions were offered, but rejected, after discussion, and the result of the deliberation of Medical Teachers is exceedingly creditable to the various delegates and the institutions which they represented. Young men who have commenced the study of medicine, and those who propose to commence, may at first look upon the anticipated changes, as imposing upon them increased labors and expenses, but a little reflection will show that it is for the better in many ways, and will, in the end, compensate for the additional outlay.

